# Impact of statin pretreatment on the complications of carotid stenting in asymptomatic patients: observational study

**DOI:** 10.1186/s12883-021-02104-z

**Published:** 2021-02-15

**Authors:** Seong Hwa Jang, Doo Hyuk Kwon, Moon-Ku Han, Hyungjong Park, Sung-Il Sohn, Huimahn Choi, Jeong-Ho Hong

**Affiliations:** 1 Department of Neurology, Keimyung University Dongsan Hospital, Keimyung University School of Medicine, 1095 Dalgubeol-daero, Dalseo-gu, Daegu, 42601 South Korea; 2 Department of Neurology, Seoul National University Bundang Hospital, Seoul National University School of Medicine, Seongnam, South Korea; 3 Department of Neurology and Neurosurgery, McGovern Medical School, University of Texas Health Science Center at Houston, Houston, TX USA

**Keywords:** Carotid stenosis, Stents, Complications, Statin

## Abstract

**Background:**

Carotid stenosis is a known risk factor for ischemic stroke, and carotid artery stenting is an effective preventive procedure. However, the stroke risk reduction for asymptomatic patients is small. Therefore, it is important to reduce the risk of complications, particularly in asymptomatic carotid stenosis. Statins are known to reduce the overall risk of periprocedural complications, although there is a lack of data focusing on asymptomatic patients. We aimed to investigate whether different doses of statin pretreatment can reduce periprocedural complications of carotid artery stenting (CAS) in patients with asymptomatic carotid artery stenosis.

**Methods:**

Between July 2003 and June 2013, 276 consecutive patients received CAS for asymptomatic carotid stenosis. Periprocedural complications included the outcome of stroke, myocardial infarction, or death within 30 days of CAS. Statin pretreatment was categorized as no-statin (*n* = 87, 31.5%), standard-dose (< 40 mg, *n* = 139, 50.4%), and high-dose statin (≥40 mg, *n* = 50, 18.1%) according to the atorvastatin equivalent dose. The Cochran-Armitage (CA) trend test was performed to investigate the association of periprocedural complications with statin dose.

**Results:**

The overall periprocedural complication rate was 3.3%. There was no significant difference in the risk of periprocedural complications between the three groups (no statin: *n* = 3 [3.4%]; standard-dose: *n* = 4 [2.9%]; high-dose *n* = 2 [4.0%] *p* = 0.923). The CA trend test did not demonstrate a trend in the proportion of periprocedural complications across increasing statin equivalent doses (*p* = 0.919).

**Conclusions:**

Statin pretreatment before CAS showed neither absolute nor dose-dependent effects against periprocedural complications in asymptomatic patients undergoing CAS.

**Supplementary Information:**

The online version contains supplementary material available at 10.1186/s12883-021-02104-z.

## Introduction

Carotid artery atheromatous disease accounts for approximately 10–20% of ischemic strokes [1, 2]. Carotid artery stenting (CAS) has grown substantially in use as an alternative definitive revascularization procedure to carotid endarterectomy (CEA) for carotid occlusive disease. In a cross-sectional analysis of US Medicare beneficiaries, the percentage of asymptomatic carotid artery stenosis among patients who underwent CAS between 1999 and 2014 was 82.5% [3]. However, the absolute risk reduction associated with CAS in patients with asymptomatic carotid artery stenosis is small [4]. Therefore, it is important to reduce periprocedural complications in asymptomatic patients with CAS.

Various treatments are available to reduce periprocedural complications [5–7]. In particular, statin therapy prior to CAS was associated with significant reductions in periprocedural risk [6–8]. In CEA, pre-statin therapy has been shown to have a protective effect only in symptomatic patients, while it was not significant in asymptomatic patients [9]. Our previous study showed that statin pretreatment reduced the incidence of periprocedural complications dose-dependently in patients who underwent CAS for symptomatic carotid artery stenosis [10]. However, this dose-effectiveness has not yet been proven in patients with asymptomatic carotid artery stenosis.

We aimed to investigate whether statin pretreatment is dose-dependently associated with a reduction of periprocedural complications in patients with asymptomatic carotid stenosis who underwent CAS.

## Methods

### Study subjects and data collection

This study used retrospective CAS registry data from two tertiary university hospitals collected between July 2003 and June 2013. We included patients with asymptomatic carotid stenosis, defined as ≥60% stenosis of the carotid artery [according to the North American Symptomatic Carotid Endarterectomy Trial criteria [11]] on digital subtraction angiography (DSA), in the absence of retinal or cerebral ischemia in the preceding 6 months. These criteria were benchmarked in the Carotid Revascularization Endarterectomy Versus Stenting Trial (CREST) [12].

We obtained clinical characteristics and procedure-related factors from our CAS registry database. All patients underwent clinical assessment at baseline, 24 h after the procedure, and daily thereafter until discharge from the hospital. The statin name and dose were obtained from hospital data. According to the lipid reduction fraction, high-dose statin was defined as atorvastatin ≥40 mg/day, and standard-dose as atorvastatin < 40 mg based on the atorvastatin-equivalent dose [13, 14]. This study was approved by the Institutional Review Board (IRB) of Keimyung University Dongsan Hospital. The requirement for informed consent was waived because of the retrospective nature of this study (IRB of Keimyung University Dongsan Hospital, IRB number 2017–05-042).

### Stenting procedure

All patients requiring CAS were treated following the current guidelines [15]. Stents and cerebral protection devices were chosen at the discretion of the neuro-interventionist. All patients received optimal medical treatment and control of vascular risk factors. Dual antiplatelet (aspirin + clopidogrel) was given routinely for at least 7 days prior to the procedure. The use of intraprocedural heparin was mandatory, with a dose of 3000 U. Dilatation after stenting was discretionary. The degree of residual stenosis at the end of the procedure was determined based on DSA.

### Outcomes and definitions

Complications after CAS, such as the rates of transient ischemic attack (TIA), stroke, myocardial infarction or death, were analyzed according to pretreatment statin dose. Periprocedural complications were considered the combined outcome of any incidents of stroke, myocardial infarction, or death within 30 days of CAS. Definitions of each periprocedural complications are derived from a previous study [10].

### Statistical analysis

Statistical variables are presented as medians (interquartile range, IQR) or frequencies (percentages). Comparative analyses between the no-statin, standard-dose, and high-dose statin groups were performed for vascular risk factors, imaging findings, and periprocedural complications. Groups were compared using chi-square tests for categorical variables, analysis of variance for parametric interval variables, or the Kruskal-Wallis test for non-parametric interval variables. We assigned the sample median for missing values of the lipid profile because we assumed that missing data were occurring randomly. Multivariable logistic regression models were created to assess the impact of variables contributing to periprocedural complications. Variables with *p* < 0.15 in the bivariate analysis were selected. In addition, the Cochran-Armitage (CA) trend test was performed to investigate the possible association of periprocedural complications with the statin dose. Statistical analyses were calculated using R statistical software (R, version 3.6.2; R project).

## Results

In our study, 276 patients were enrolled. The majority were male (83.7%), and the median age was 70.0 years (IQR, 64.0–74.0). Most patients had > 70% stenosis (95.6%). The most common vascular risk factor was hypertension (66.7%), followed by diabetes mellitus (42.4%), smoking (42.4%), and hyperlipidemia (33.7%). Prior to CAS, all patients were on antiplatelet treatment. The patients were allocated into three groups according to the atorvastatin equivalent dose: no-statin (*n* = 87, 31.5%), standard-dose (*n* = 139, 50.4%), and high-dose statin (*n* = 50, 18.1%). Overall baseline characteristics are presented in Table 1.

**Table 1:** Baseline characteristics of patients undergoing stenting for asymptomatic carotid artery stenosis

	Total (*N* = 276)	No-statin (*N* = 87)	Standard-dose (*N* = 139)	High-dose (*N* = 50)	*p*-value
Age, years	70.0 (64.0–74.0)	70.0 (63.5–74.0)	70.0 (63.5–74.0)	69.0 (64.0–72.0)	0.417
Sex, male	231 (83.7%)	78 (89.7%)	114 (82.0%)	39 (78.0%)	0.154
Vascular risk factors					
Hypertension	184 (66.7%)	52 (59.8%)	96 (69.1%)	36 (72.0%)	0.239
Diabetes mellitus	117 (42.4%)	33 (37.9%)	62 (44.6%)	25 (50.0%)	0.363
Hyperlipidemia	93 (33.7%)	12 (13.8%)	63 (45.3%)	18 (36.0%)	< 0.001
Ischemic heart disease	51 (18.5%)	11 (12.6%)	28 (20.1%)	12 (24.0%)	0.198
Atrial fibrillation	13 (4.7%)	3 (3.4%)	6 (4.3%)	4 (8.0%)	0.458
Smoking	117 (42.4%)	35 (40.2%)	63 (45.3%)	19 (38.0%)	0.591
Total cholesterol	166.0 (140.0–194.0)	181.0 (151.0–199.0)	164.0 (135.5–192.0)	152.0 (135.0–178.0)	0.008
Triglyceride	121.0 (88.0–165.5)	120.5 (87.2–159.3)	114.3 (83.9–155.5)	145.5 (113.5–177.5)	0.048
High-density lipoprotein	41.0 (35.0–49.0)	42.0 (35.0–49.0)	41.0 (35.0–49.2)	40.0 (36.0–47.5)	0.954
Low-density lipoprotein	91.0 (68.0–114.5)	98.0 (81.0–118.9)	88.5 (66.0–113.0)	71.0 (57.5–113.2)	0.048
Pre-stenting antiplatelet	276 (100%)	87 (100%)	139 (100%)	50 (100%)	1.000
Degree of stenosis					0.207
60–69%	12 (4.4%)	4 (4.6%)	7 (5.0%)	1 (2.0%)	
70–89%	195 (70.6%)	55 (63.2%)	99 (71.2%)	41 (82.0%)	
90–99%	69 (25.0%)	28 (32.2%)	33 (23.7%)	8 (16.0%)	
Residual stenosis	10.0 (0.0–29.2)	5.0 (0.0–20.0)	10.0 (0.0–30.0)	20.0 (7.0–30.0)	0.003
Pre-stenting dilatation	266 (96.4%)	81 (93.1%)	137 (98.6%)	48 (96.0%)	0.101
Post-stenting dilatation	106 (38.4%)	27 (31.0%)	58 (41.7%)	21 (42.0%)	0.232

Values are presented as percentage or median (interquartile range)

The groups according to statin dose did not differ significantly in age. In procedure-related characteristics, such as pre-stenting dilatation and post-stent dilatation, there was no difference between the three groups. Although the degree of stenosis was similar between the three groups (*p* = 0.207), residual stenosis was significantly higher in the high-dose statin group compared to other groups (*p* = 0.003). In terms of lipid profile, there were no significant differences between groups in high-density lipoproteins. However, the high-dose statin group had lower low-density lipoproteins (LDL) and total cholesterol than other groups. A summary of statin drugs and doses used is shown in Additional file 1: Supplementary Table I.

Overall, the periprocedural complication rate was 3.3% (9 cases). The rate for any type of stroke or death was 3.0% (8 cases). Six patients (2.2%) had ischemic strokes, two (0.7%) had hemorrhagic strokes, and one (0.3%) had a myocardial infarction (Table 2). Considering the degree of stenosis, one periprocedural complication (1/12, 8.3%) occurred in a patient with 60 to 69% stenosis, six complications (6/195, 3.1%) occurred in those with 70 to 89% stenosis, and two complications (2/69, 2.9%) occurred in those with 90 to 99% stenosis. Analyzing results according to pretreatment statin dose, patients in the high-dose statin group had two complications, the standard-dose statin group had four complications, and the no-statin group had three complications. This difference was not statistically significant (*p* = 0.923). When analyzed depending on the use of pretreatment statin, no difference in periprocedural complications was found between the groups (Additional file 1: Supplementary Table II). After adjusting for confounders, including hyperlipidemia, residual stenosis, and pre-stenting dilatation, statin-pretreatment remained a non-independent factor associated with periprocedural complications after CAS (Standard-dose statin: odds ratio, 1.003; 95% CI [0.953, 1.055]; High-dose statin: odds ratio, 1.012; 95% CI [0.950, 1.079]) (Table 3). The CA trend test also did not demonstrate a trend in the proportion of periprocedural complications across increasing statin equivalent dose (*p* = 0.919).

**Table 2:** Periprocedural complications within 30 days of stenting for asymptomatic carotid artery stenosis

	Total (*N* = 276)	No-statin (*N* = 87)	Standard-dose (*N* = 139)	High-dose (*N* = 50)	*p*-value
Periprocedural complications	9 (3.3%)	3 (3.4%)	4 (2.9%)	2 (4.0%)	0.923
Ischemic stroke	6 (2.2%)	1 (1.1%)	4 (2.9%)	1 (2.0%)	0.684
Hemorrhage stroke	2 (0.7%)	1 (1.1%)	0 (0.0%)	1 (2.0%)	0.307
^a^Myocardial infarction	1 (0.30%)	1 (1.1%)	0 (0.0%)	0 (0.0%)	0.336
Death	0 (0.0%)	0 (0.0%)	0 (0.0%)	0 (0.0%)	1.000

^a^One patient had myocardial infarction after 1 day

**Table 3:** Multivariable logistic regression for complications within 30 days of stenting for asymptomatic carotid artery stenosis

	Odds Ratio	95% Confidence Interval	*p*-value
Hyperlipidemia	0.967	0.922–1.013	0.163
Residual stenosis	0.999	0.998–1.001	0.894
Pre-stenting dilatation	1.032	0.920–1.158	0.588
Statin dose			
No statin	Reference	Reference	Reference
Standard-dose statin	1.003	0.953–1.055	0.895
High-dose statin	1.012	0.950–1.079	0.695

## Discussion

In our study, the CAS 30-day periprocedural complication rate was 3.3% (9 of 276), which is consistent with previous studies on asymptomatic carotid artery stenosis [4, 12]. The overall periprocedural complications were not different between the three groups according to the pretreatment statin dose, and showed no linear trend of association to the statin dose. In addition, when the analysis was conducted comparing use to no-use of statin before CAS, there was no difference in the results.

Statins are considered an option for preventing periprocedural complications. Pretreatment statin use has been shown by previous studies to significantly decrease the frequency of cardiovascular, cerebrovascular, and secondary stroke events after vascular procedures or endarterectomy [9, 16–22]. This might be due to the multiple effects of statin, such as plaque stabilization and reduction of intravascular thrombosis. Preventive effects of statins are supported by findings of its use being associated with reduced plaque volume and atheroma regression in the carotid circulation, [23] in addition to less embolic debris during CAS [24]. However, all of these studies included both symptomatic and asymptomatic patients [25]. There is no study about the pretreatment statin effects on periprocedural complications of CAS limited to asymptomatic patients. In contrast to studies with symptomatic carotid artery stenosis, our study demonstrated no significant dose-dependent or absolute statin effects in preventing periprocedural complications in asymptomatic patients with carotid artery stenosis. There is a possible explanation for this finding. A previous study suggests that infiltration of the fibrous cap with foam cells, fibrous cap thinning, and plaque rupture are more common in patients with symptomatic carotid artery stenosis than in asymptomatic patients [26]. Similar to acute coronary syndromes, plaque ruptures are an important etiopathogenetic factor of neurological deterioration as a result of carotid artery stenosis. During stent deployment, embolism from the carotid artery plaque is generally responsible for the majority of new ischemic lesions. Based on this study, the multiple pleiotropic effects of statins did not alter the risk of periprocedural complications in asymptomatic patients with carotid artery stenosis because the plaques are generally more stable. Nonetheless, caution is advised in the interpretation of our results, which showed that there is no protective effect of statin, limited to the periprocedural period. Statins are known to have protective effects on long-term outcome [6, 27].

In this study, we observed an LDL-lowering effect of statins. However, statins have multiple pleiotropic effects, such as promoting endothelial function, reducing inflammation, affecting arterial myocyte proliferation, migration and apoptosis, as well as regulating platelet activity, plaque stability, and the coagulation process. Therefore, LDL is not the only concern when contemplating successful risk reduction [28, 29]. As previous studies suggest, statin treatment should be administered in accordance with the global vascular risk factors and not chiefly according to base LDL level [30, 31]. Consistently, our results indicated that LDL levels alone did not correlate with the risk of periprocedural complications.

Our study has several limitations. First, this was a retrospective study with a relatively small sample, which did not allow us to extract statistically significantly results in the analysis of periprocedural complications (9 cases). Second, there was a lack of data on pretreatment duration. Thus, it was not possible to evaluate where this parameter was associated with periprocedural complications. Third, we lacked information on the type of stents used. Previous studies have shown a higher risk of periprocedural stroke in patients treated with open-cell stent devices compared to those implanted with closed-cell devices [32, 33]. Fourth, we cannot be certain that there were no differences in the original plaque morphology or vascular anatomy of the groups considered, which might have differentially influenced the risk of periprocedural complications. Therefore, our results should be verified in randomized trials or prospective trials with larger datasets.

In conclusion, this study shows that statin pretreatment did not have dose-dependent or absolute effects on periprocedural complications risk in patients undergoing CAS for the treatment of asymptomatic carotid artery stenosis.

## Supplementary Information

**Additional file 1: Supplementary Table 1**. Summary of statin drug and dose use before carotid artery stenting. **Supplementary Table 2**. Periprocedural complications within 30 days of stenting for asymptomatic carotid artery stenosis.

## Data Availability

The datasets generated during and/or analyzed during the current study are available from the corresponding author on reasonable request.

## References

[1] Young KC, Jain A, Jain M, Replogle RE, Benesch CG, Jahromi BS (2011). Evidence-based treatment of carotid artery stenosis. Neurosurg Focus.

[2] White H, Boden-Albala B, Wang C, Elkind MS, Rundek T, Wright CB (2005). Ischemic stroke subtype incidence among whites, blacks, and Hispanics: the northern Manhattan study. Circulation..

[3] Lichtman JH, Jones MR, Leifheit EC, Sheffet AJ, Howard G, Lal BK (2017). Carotid endarterectomy and carotid artery stenting in the US Medicare population, 1999-2014. JAMA..

[4] Rosenfield K, Matsumura JS, Chaturvedi S, Riles T, Ansel GM, Metzger DC (2016). Randomized trial of stent versus surgery for asymptomatic carotid stenosis. N Engl J Med.

[5] Naylor AR, Ricco JB, de Borst GJ, Debus S, de Haro J, Halliday A (2018). Editor's choice - Management of Atherosclerotic Carotid and Vertebral Artery Disease: 2017 clinical practice guidelines of the European Society for Vascular Surgery (ESVS). Eur J Vasc Endovasc Surg.

[6] Verzini F, De Rango P, Parlani G, Giordano G, Caso V, Cieri E (2011). Effects of statins on early and late results of carotid stenting. J Vasc Surg.

[7] Reiff T, Amiri H, Rohde S, Hacke W, Ringleb PA (2014). Statins reduce peri-procedural complications in carotid stenting. Eur J Vasc Endovasc Surg.

[8] Groschel K, Ernemann U, Schulz JB, Nagele T, Terborg C, Kastrup A (2006). Statin therapy at carotid angioplasty and stent placement: effect on procedure-related stroke, myocardial infarction, and death. Radiology..

[9] Kennedy J, Quan H, Buchan AM, Ghali WA, Feasby TE (2005). Statins are associated with better outcomes after carotid endarterectomy in symptomatic patients. Stroke..

[10] Hong JH, Sohn SI, Kwak J, Yoo J, Chang HW, Kwon OK (2017). Dose-dependent effect of statin pretreatment on preventing the Periprocedural complications of carotid artery stenting. Stroke..

[11] North American Symptomatic Carotid Endarterectomy Trial (1991). Methods, patient characteristics, and progress. Stroke..

[12] Brott TG, Hobson RW, Howard G, Roubin GS, Clark WM, Brooks W (2010). Stenting versus endarterectomy for treatment of carotid-artery stenosis. N Engl J Med.

[13] Law MR, Wald NJ, Rudnicka AR (2003). Quantifying effect of statins on low density lipoprotein cholesterol, ischaemic heart disease, and stroke: systematic review and meta-analysis. BMJ..

[14] Jones P, Kafonek S, Laurora I, Hunninghake D (1998). Comparative dose efficacy study of atorvastatin versus simvastatin, pravastatin, lovastatin, and fluvastatin in patients with hypercholesterolemia (the CURVES study). Am J Cardiol.

[15] Brott TG, Halperin JL, Abbara S, Bacharach JM, Barr JD, Bush RL (2011). 2011 ASA/ACCF/AHA/AANN/AANS/ACR/ASNR/CNS/SAIP/SCAI/SIR/SNIS/SVM/SVS guideline on the management of patients with extracranial carotid and vertebral artery disease. Stroke..

[16] Chan AW, Bhatt DL, Chew DP, Quinn MJ, Moliterno DJ, Topol EJ (2002). Early and sustained survival benefit associated with statin therapy at the time of percutaneous coronary intervention. Circulation..

[17] Pasceri V, Patti G, Nusca A, Pristipino C, Richichi G, Di Sciascio G (2004). Randomized trial of atorvastatin for reduction of myocardial damage during coronary intervention: results from the ARMYDA (atorvastatin for reduction of MYocardial damage during angioplasty) study. Circulation..

[18] McGirt MJ, Perler BA, Brooke BS, Woodworth GF, Coon A, Jain S (2005). 3-hydroxy-3-methylglutaryl coenzyme a reductase inhibitors reduce the risk of perioperative stroke and mortality after carotid endarterectomy. J Vasc Surg.

[19] Sillesen H, Amarenco P, Hennerici MG, Callahan A, Goldstein LB, Zivin J (2008). Atorvastatin reduces the risk of cardiovascular events in patients with carotid atherosclerosis: a secondary analysis of the stroke prevention by aggressive reduction in cholesterol levels (SPARCL) trial. Stroke..

[20] Amarenco P, Labreuche J (2009). Lipid management in the prevention of stroke: review and updated meta-analysis of statins for stroke prevention. Lancet Neurol.

[21] Antoniou GA, Hajibandeh S, Hajibandeh S, Vallabhaneni SR, Brennan JA, Torella F (2015). Meta-analysis of the effects of statins on perioperative outcomes in vascular and endovascular surgery. J Vasc Surg.

[22] Schouten O, Boersma E, Hoeks SE, Benner R, van Urk H, van Sambeek MR (2009). Fluvastatin and perioperative events in patients undergoing vascular surgery. N Engl J Med.

[23] Migrino RQ, Bowers M, Harmann L, Prost R, LaDisa JF (2011). Carotid plaque regression following 6-month statin therapy assessed by 3T cardiovascular magnetic resonance: comparison with ultrasound intima media thickness. J Cardiovasc Magn Reson.

[24] Tadros RO, Vouyouka AG, Chung C, Malik RK, Krishnan P, Ellozy SH (2013). The effect of statin use on embolic potential during carotid angioplasty and stenting. Ann Vasc Surg.

[25] Texakalidis P, Giannopoulos S, Jonnalagadda AK, Chitale RV, Jabbour P, Armstrong EJ (2018). Preoperative use of statins in carotid artery stenting: a systematic review and meta-analysis. J Endovasc Ther.

[26] Carr S, Farb A, Pearce WH, Virmani R, Yao JS (1996). Atherosclerotic plaque rupture in symptomatic carotid artery stenosis. J Vasc Surg.

[27] Hussain MA, Saposnik G, Raju S, Salata K, Mamdani M, Tu JV (2018). Association between statin use and cardiovascular events after carotid artery revascularization. J Am Heart Assoc.

[28] Perler BA (2007). Should statins be given routinely before carotid endarterectomy?. Perspect Vasc Surg Endovasc Ther.

[29] de Lorenzo F, Feher M, Martin J, Collot-Teixeira S, Dotsenko O, McGregor JL (2006). Statin therapy-evidence beyond lipid lowering contributing to plaque stability. Curr Med Chem.

[30] Heart Protection Study Collaborative G (2002). MRC/BHF heart protection study of cholesterol lowering with simvastatin in 20,536 high-risk individuals: a randomised placebo-controlled trial. Lancet..

[31] Collins R, Armitage J, Parish S, Sleight P, Peto R (2004). Heart protection study collaborative G. effects of cholesterol-lowering with simvastatin on stroke and other major vascular events in 20536 people with cerebrovascular disease or other high-risk conditions. Lancet..

[32] Doig D, Turner EL, Dobson J, Featherstone RL, Lo RT, Gaines PA (2016). Predictors of stroke, myocardial infarction or death within 30 days of carotid artery stenting: results from the international carotid stenting study. Eur J Vasc Endovasc Surg.

[33] Jansen O, Fiehler J, Hartmann M, Bruckmann H (2009). Protection or nonprotection in carotid stent angioplasty: the influence of interventional techniques on outcome data from the SPACE trial. Stroke..

