# Supplementary material for: Impact of statin pretreatment on the complications of carotid stenting in asymptomatic patients: observational study
Source: BMC Neurol. 2021 Feb 15;21:75. doi: 10.1186/s12883-021-02104-z (PMC7883458; doi:10.1186/s12883-021-02104-z)
Supplement: Supplementary file 1 — Additional file 1: Supplementary Table 1. Summary of statin drug and dose use before carotid artery stenting. Supplementary Table 2. Periprocedural complications within 30 days of stenting for asymptomatic carotid artery stenosis. [file 12883_2021_2104_MOESM1_ESM.docx]

**Impact of statin pretreatment on the complications of carotid stenting in asymptomatic patients : observational study**

Seong Hwa Jang, MD^1^, Doo Hyuk Kwon, MD^1^, Moon-Ku Han, MD, PhD^2^, Hyungjong Park, MD^1^, Sung-Il Sohn, MD, PhD^1^, Huimahn Choi, MD^3^, Jeong-Ho Hong, MD, PhD^1^

^1^Department of Neurology, Keimyung University Dongsan Hospital, Keimyung University School of Medicine, Daegu, South Korea

^2^Department of Neurology, Seoul National University Bundang Hospital, Seoul National University School of Medicine, Seongnam, South Korea

^3^Department of Neurology and Neurosurgery, McGovern Medical School, University of Texas Health Science Center at Houston, TX, USA

**Corresponding author:**

Jeong-Ho Hong, MD, PhD

Department of Neurology, Keimyung University School of Medicine

1095 Dalgubeol-daero, Dalseo-gu, Daegu 42601, Korea

Tel: +82-53-258-4379

Fax: +82-53-258-4380

Email: neurohong79@gmail.com

**SUPPLEMENTAL MATERIAL**

**Supplementary Table I. Summary of statin drug and dose use before carotid artery stenting.**

| Atorvastatin  Equivalent dose | Total number  (N = 189) | Atorvastatin  (N = 150) | Rosuvastatin  (N = 14) | Simvastatin  (N = 11) | Fluvastatin  (N = 1) | Lovastatin  (N = 1) | Pravastatin  (N = 6) | Pitavastatin  (N = 6) |
| --- | --- | --- | --- | --- | --- | --- | --- | --- |
| 5 mg | 6 |  |  |  | 40 mg (1) | 20 mg (1) | 10 mg (4) |  |
| 10 mg | 96 | 77 |  | 20 mg  (11) |  |  | 40 mg (2) | 2 mg  (6) |
| 20 mg | 37 | 37 |  |  |  |  |  |  |
| 40 mg | 32 | 23 | 10 mg  (9) |  |  |  |  |  |
| 80 mg | 18 | 13 | 20 mg  (5) |  |  |  |  |  |

**Supplementary Table II. Periprocedural complications within 30 days of stenting for asymptomatic carotid artery stenosis**

|  | No statin (N = 87) | Statin (N = 189) | *p*-value |
| --- | --- | --- | --- |
| Periprocedural complications | 3 (3.4 %) | 6 (3.2 %) | 1.000 |
| Ischemic stroke | 1 (1.1 %) | 5 (2.6 %) | 0.728 |
| Hemorrhage stroke | 1 (1.1 %) | 1 (0.5 %) | 1.000 |
| *Myocardial infarction | 1 (1.1%) | 0 (0.0%) | 0.690 |
| Death | 0 (0.0%) | 0 (0.0%) | 1.000 |

*One patient had myocardial infarction after 1 day.
